# Early Prediction of Disease Progression in Patients with Severe COVID-19 Using C-Reactive Protein to Albumin Ratio

**DOI:** 10.1155/2021/6304189

**Published:** 2021-12-03

**Authors:** Yi Li, Haitao Li, Chao Song, Rongli Lu, Yuhao Zhao, Fengyu Lin, Duoduo Han, Lingli Chen, Pinhua Pan, Minhui Dai

**Affiliations:** ^1^The Department of Respiratory Medicine, Xiangya Hospital, Central South University, Changsha 410008, China; ^2^First Department of Thoracic Medicine, Hunan Cancer Hospital/The Affiliated Cancer Hospital of Xiangya School of Medicine, Central South University, Changsha 410008, China; ^3^Nosocomial Infection Control Center, Xiangya Hospital, Central South University, Changsha 410008, China; ^4^National Clinical Research Center for Geriatric Disorders, Xiangya Hospital, Central South University, Changsha, Hunan 410008, China

## Abstract

**Background:**

Early identification of patients with severe coronavirus disease (COVID-19) at an increased risk of progression may promote more individualized treatment schemes and optimize the use of medical resources. This study is aimed at investigating the utility of the C-reactive protein to albumin (CRP/Alb) ratio for early risk stratification of patients.

**Methods:**

We retrospectively reviewed 557 patients with COVID-19 with confirmed outcomes (discharged or deceased) admitted to the West Court of Union Hospital, Wuhan, China, between January 29, 2020 and April 8, 2020. Patients with severe COVID-19 (*n* = 465) were divided into stable (*n* = 409) and progressive (*n* = 56) groups according to whether they progressed to critical illness or death during hospitalization. To predict disease progression, the CRP/Alb ratio was evaluated on admission.

**Results:**

The levels of new biomarkers, including neutrophil-to-lymphocyte ratio, platelet-to-lymphocyte ratio, CRP/Alb ratio, and systemic immune-inflammation index, were higher in patients with progressive disease than in those with stable disease. Correlation analysis showed that the CRP/Alb ratio had the strongest positive correlation with the sequential organ failure assessment score and length of hospital stay in survivors. Multivariate logistic regression analysis showed that percutaneous oxygen saturation (SpO2), D-dimer levels, and the CRP/Alb ratio were risk factors for disease progression. To predict clinical progression, the areas under the receiver operating characteristic curves of Alb, CRP, CRP/Alb ratio, SpO2, and D-dimer were 0.769, 0.838, 0.866, 0.107, and 0.748, respectively. Moreover, patients with a high CRP/Alb ratio (≥1.843) had a markedly higher rate of clinical deterioration (log − rank *p* < 0.001). A higher CRP/Alb ratio (≥1.843) was also closely associated with higher rates of hospital mortality, ICU admission, invasive mechanical ventilation, and a longer hospital stay.

**Conclusion:**

The CRP/Alb ratio can predict the risk of progression to critical disease or death early, providing a promising prognostic biomarker for risk stratification and clinical management of patients with severe COVID-19.

## 1. Introduction

The coronavirus disease-2019 (COVID-19) pandemic caused by severe acute respiratory syndrome coronavirus 2 (SARS-CoV-2) has seriously threatened global public health security. Although vaccines are now accepted worldwide, we still could not control COVID-19. As of November 7, 2021, the overall number of confirmed cases of COVID-19 was 249,425,563, and that of reported deaths was 5,042,690 worldwide according to the World Health Organization (WHO) report [1]. Previous studies have shown that about 80% of COVID-19 cases are mild or moderate, with recovery with or without hospitalization treatment, while some severe COVID-19 cases, especially among older patients and those with several pre-existing comorbidities, are prone to progression to critical illness requiring intensive care or death [2, 3]. In the face of this rapidly spreading and progressive infectious disease, early identification of patients with severe COVID-19 who are at elevated risk of worsening disease progression may promote more individualized treatment schemes and optimize the allocation of limited medical resources. Consequently, novel predictive biomarkers for disease progression from severe disease to critical disease or death are urgently needed.

Clinical research has proven that an excessive inflammatory response caused by SARS-CoV-2 and a subsequent out-of-control cytokine storm are important causes of clinical deterioration and death in patients with severe COVID-19 [4, 5]. In the acute phase of inflammation, C-reactive protein (CRP) production increases, while albumin (Alb) production decreases [6]. Elevated CRP levels and hypoproteinemia are not only the key indicators of disease severity but also the risk factors for death in patients with severe COVID-19 and are indicative of a cytokine storm, a common occurrence in patients with COVID-19 [7, 8]. Based on these characteristics, clinical researchers speculate that the CRP/Alb ratio, a compound index, is a promising new biomarker in patients with cytokine storm syndrome. Previous studies have shown that CRP/Alb ratio is a promising prognostic biomarker for critically illness [9], cancers [10, 11], and Kawasaki disease [12]. Based on current data, CRP/Alb ratio could predict adverse clinical outcomes. Recently, studies with small sample sizes have shown a significant increase in the CRP/Alb ratio in patients with severe COVID-19 and nonsurvivors [13, 14]. However, its correlation with the risk of clinical progression and deterioration in patients with severe COVID-19 has rarely been reported. In this retrospective study, we aimed to analyze the demographics and baseline clinical and laboratory characteristics of patients with progressive COVID-19 and those with stable COVID-19 and evaluate the capability of CRP/Alb ratio in predicting the risk of progression to critical illness or death in the early stages of severe COVID-19.

## 2. Materials and Methods

### 2.1. Study Design and Participants

**Figure 1:**
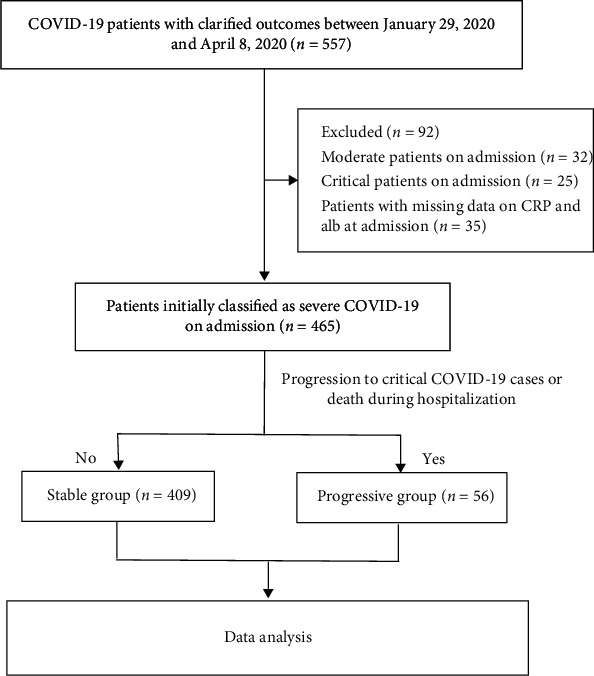
Flow diagram of the study population.

This retrospective, single-center, observational study included patients with severe COVID-19 who were admitted to the West Court of Union Hospital of Huazhong University of Science and Technology during management by the national medical team from January 29, 2020 to April 8, 2020 [15]. This hospital was used as an intensive care center at the peak of the COVID-19 pandemic in Wuhan. All patients were diagnosed with COVID-19 based on the WHO Interim Guidelines. We excluded patients with moderate or critical COVID-19 and those with missing CRP and Alb data at admission. A total of 465 patients with severe COVID-19 with a clarified clinical outcome (died or recovered) were enrolled in this study, and the study flow diagram is shown in Figure 1. The primary study endpoint was the progression of severe COVID-19 to critical disease or death during hospitalization and other clinical outcomes, including intensive care unit (ICU) admission, need for invasive mechanical ventilation, and discharge. This study was approved by the Ethics Committee of Union Hospital of Huazhong University of Science and Technology and the Ethics Board of Xiangya Hospital, Central South University (No. 202003049). The requirement for written informed consent was waived by the Ethics Commission of the designated hospital because of the infectious nature of the disease.

### 2.2. Definitions

The clinical types of COVID-19 were classified according to the Diagnosis and Treatment Protocol for Novel Coronavirus Pneumonia (Trial Version 7) published by the National Health Commission and National Administration of Traditional Chinese Medicine of China [16]. Patients were classified as having severe COVID-19 if they met any of the following criteria: (1) respiratory rate ≥ 30 breaths per minute, (2) finger oxygen saturation ≤ 93% at rest, and (3) PaO2/FiO2 ≤ 300 mmHg. Patients were classified as having critical COVID-19 cases if they met any of the following criteria: (1) respiratory failure requiring mechanical ventilation, (2) shock, and (3) other organ failures requiring ICU care.

According to the clinical outcomes during hospitalization, patients with severe COVID-19 were divided into progressive and stable groups. Patients with severe COVID-19 who progressed to critical disease or death were categorized into the progressive group, while those with severe COVID-19 who recovered and were discharged from the hospital, and those who did not progress to critical disease were categorized into the stable group. The new serological markers and their formulas are as follows: (1) systemic immune − inflammation index (SII) = platelet count (10^9^/L) × neutrophil count (10^9^/L)/lymphocyte count (10^9^/L) [17] and (2) prognostic nutritional index (PNI) = 10 × albumin (g/L) + 5 × lymphocyte count (10^9^/L) [18].

### 2.3. Data Collection

The clinical records and laboratory data of each patient were obtained from the electronic medical system. A group of experienced respiratory clinicians reviewed and refined the data. Data on demographics, comorbidities, clinical symptoms and signs, and clinical outcomes (progression of severe COVID-19 to critical disease or death during hospitalization) were extracted from the patients' electronic medical records. Data on laboratory test findings at admission, including those for CRP, Alb, liver function, kidney function, coagulation function, and routine blood parameters, were collected and evaluated. Disease severity was evaluated using the CURB-65 and sequential organ failure assessment (SOFA) scores.

### 2.4. Laboratory Tests

A BC-6800 automatic hematology analyzer (Mindray, Shenzhen, China) was used for routine blood parameter analysis. Serum biochemical parameters, including liver function parameters, renal function index, lipid profile, Alb, and CRP, were tested on a LABOSPECT 008 AS automatic biochemical analyzer using the manufacturer's reagents (Hitachi, Tokyo, Japan). CRP levels were detected by immunoturbidimetry while albumin levels were detected using the bromocresol green method. The reference levels for serum albumin and CRP were 33-55 g/L and 0-8 mg/L, respectively. Coagulation function parameters were assayed using an SF-8100 automated blood coagulation analyzer (Succeeder, Beijing, China). All laboratory data were tested in the same laboratory using standardization and certification procedures.

### 2.5. Statistical Analysis

Categorical variables, presented as numbers (percentages), were compared using the chi-square test or Fisher's exact test. Continuous variables with normal distribution, presented as mean ± standard deviation, were compared using Student's *t*-test, whereas continuous variables with skewed distribution, presented as medians (interquartile ranges), were compared using the Mann–Whitney *U* test. Correlations between variables were analyzed using Spearman's coefficient. Receiver operating characteristic curve analyses were performed to determine the cutoff values, sensitivity, and specificity of biomarkers for predicting clinical disease progression. The best Youden index (sensitivity + specificity -1) was obtained to calculate the appropriate cutoff point for potential mediators to predict disease progression and deterioration. In addition, risk factors were evaluated using univariate analysis, and variables with statistical significance in the univariate analysis were included in the multivariate logistic regression analysis using forward stepwise selection based on the likelihood ratio to calculate independent risk factors. Differences in the clinical disease progression of patients with severe COVID-19 were compared using Kaplan-Meier analysis with the log-rank test. For all analyses, *p* < 0.05 (two-tailed) was considered statistically significant. GraphPad Prism 8.0 (GraphPad Software, La Jolla, CA, USA), SPSS software (version 25; SPSS Inc., Chicago, IL, USA), and MedCalc Statistical Software (Mariakerke, Belgium) were used for statistical graphs and analyses.

## 3. Results

### 3.1. Demographics and Baseline Clinical Characteristics of Patients with Progressive and Stable COVID-19

A total of 465 patients with severe COVID-19 were included in the final analysis according to the selection criteria (Figure 1). During hospitalization, 56/465 (12.0%) patients progressed to critical COVID-19 or death. The clinical characteristics and outcomes of patients who progressed to critical COVID-19 or death (progressive group, *n* = 56) and patients who did not (stable group, *n* = 409) are summarized in Table 1. The mean age of the 465 patients was 62.0 years, and 248 (53.3%) patients were men. Hypertension was the most common comorbidity, followed by diabetes, coronary heart disease, chronic obstructive pulmonary disease, and malignancy. Compared to the stable group, the progressive group had a higher proportion of men (71.4% vs. 50.9%, *p* = 0.004) and patients with hypertension (69.7% vs. 45.0%, *p* = 0.001). The respiration rate and temperature were significantly higher in the progressive group than in the stable group. Patients in the progressive group had higher rates of in-hospital death, ICU admission, invasive mechanical ventilation, higher disease severity score, and a longer hospital stay than those in the stable group (Table 1). Compared to stable patients, progressive patients had significantly higher counts of white blood cells and neutrophils, higher levels of aspartate aminotransferase, lactate dehydrogenase, total bilirubin, blood urea nitrogen, creatinine, creatine kinase, high-sensitive cardiac troponin I, CRP, D-dimer, and procalcitonin, lower counts of lymphocytes and platelets; and lower levels of albumin, high-density lipoprotein cholesterol, and apolipoprotein A-I (Table 2).

### 3.2. Novel Biomarkers for Disease Progression and Severity

**Figure 2:**
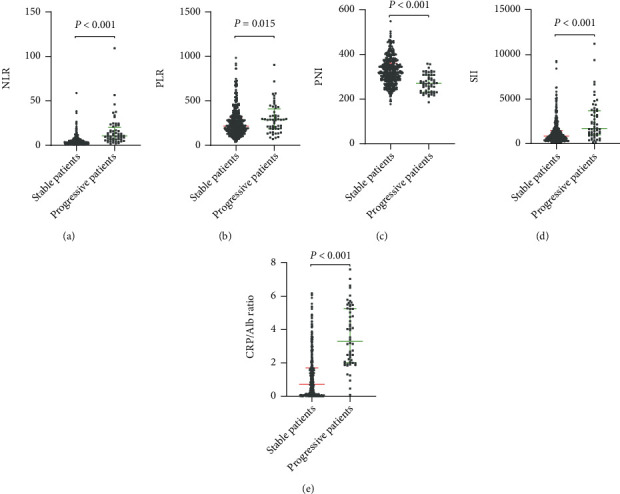
Novel serological indicators between patients with stable and progressive COVID-19. Data are presented as medians (IQR). Statistical significance was calculated by Mann–Whitney *U* test. *p* values indicate differences between patients with stable and progressive COVID-19. Abbreviations: NLR: neutrophil-lymphocyte ratio; PLR: platelet-lymphocyte ratio; PNI: prognostic nutritional index; SII: systemic immune-inflammation index; CRP: C-reactive protein; Alb: albumin.

The levels of new biomarkers, including neutrophil-to-lymphocyte ratio (NLR) (10.60 [6.53–20.10] vs. 3.58 [2.22–5.88], *p* < 0.001), platelet-to-lymphocyte ratio (PLR) (286.65 [185.85–409.03] vs. 215.79 [154.30-310.63], *p* = 0.015), systemic immune-inflammation index (SII) (1700.04 [979.15–3710.90] vs. 847.30 [478.24–1468.94], *p* < 0.001), and CRP/Alb ratio (3.29 [2.02–5.26] vs. 0.72 [0.10–1.70], *p* < 0.001), were higher in patients with progressive disease than in those with stable disease (Figure 2). Patients with progressive disease had lower prognostic nutritional index (PNI) values than those with stable disease (271.20 [232.83–309.33] vs. 321.90 [286.95–362.33], *p* < 0.001, Figure 2(c)). Correlation analysis was performed to correlate disease severity scores and the length of hospital stay with the new biomarkers. As shown in Table 3, NLR, PLR, SII, and CRP/Alb ratio were positively correlated with the SOFA scores, CURB-65 scores, and length of hospital stay in survivors (*p* ≤ 0.001). PNI was inversely correlated with the disease severity scores and length of hospital stay among survivors (*p* < 0.001). The CRP/Alb ratio had the strongest positive correlation with the SOFA score (*r*_*s*_ = 0.661, *p* < 0.001) and length of stay (*r*_*s*_ = 0.552, *p* < 0.001) in survivors (Table 3).

### 3.3. Risk Factors for Disease Progression in Patients with Severe COVID-19

Univariate logistic analysis of the risk factors for progression in patients with severe COVID-19 based on demographic factors and clinical laboratory test indicators at admission revealed that age, male sex, comorbidities (hypertension, coronary heart disease, and chronic obstructive lung disease), respiratory rate, SpO_2_,temperature, NLR, PLR, PNI, SII, the CRP/Alb ratio, aspartate aminotransferase levels, lactate dehydrogenase levels, total bilirubin levels, creatine kinase levels, blood urea nitrogen levels, D-dimer levels, and prothrombin time were significantly associated with disease progression (Table 4). Twenty significant factors in the univariate analysis were included in the multivariate stepwise regression analysis. Multivariate logistic regression analysis showed that SpO_2_ (OR: 0.794, 95% CI: 0.726-0.868, *p* = 0.030), D-dimer levels (OR: 1.219, 95% CI: 1.085-1.370, *p* = 0.001), and CRP/Alb ratio (OR: 1.888, 95% CI: 1.516-2.352, *p* < 0.001) were risk factors for disease progression in the final model (Table 4).

### 3.4. Diagnostic Value and Predictors of Disease Progression and Clinical Outcomes

**Figure 3:**
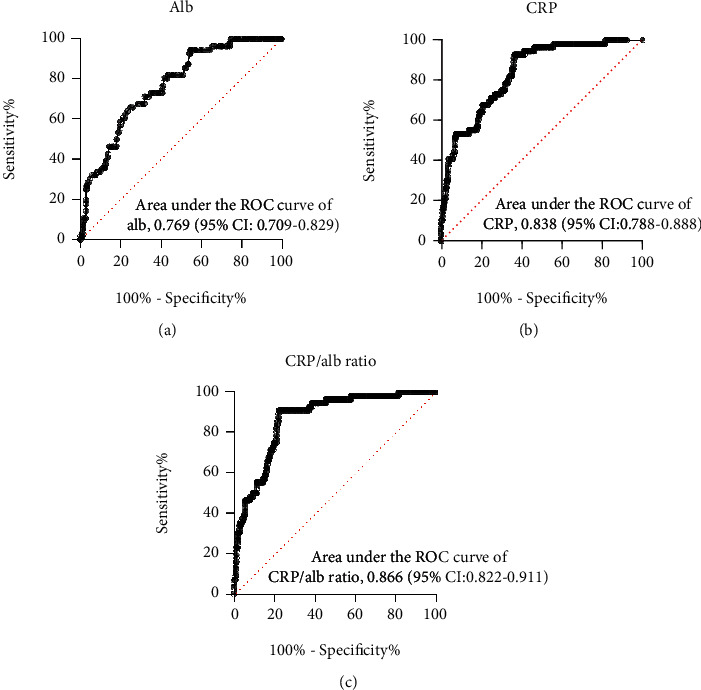
Receiver operating characteristic curves of (a) albumin, (b) CRP, and (c) CRP/Alb ratio for the prediction of disease severity progression. Abbreviations: CRP: C-reactive protein; Alb: albumin.

**Figure 4:**
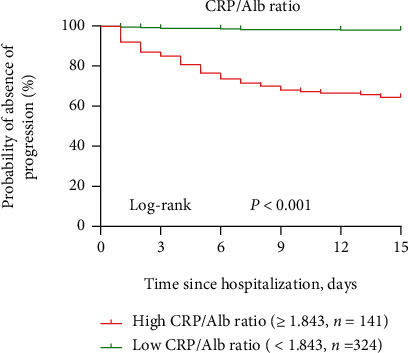
Kaplan-Meier curves of postadmission time until severe clinical progression in patients with severe COVID-19 patients with CRP/Alb ratios at admission. A log-rank test was used to evaluate differences between groups. The disease progression rates were 35.5% (50 of 141) and 1.9% (6 of 324) for the high CRP/Alb ratio(≥1.843) group and low CRP/Alb ratio (<1.843) group, respectively, at the observed endpoint (progression to critical COVID-19 cases), respectively (*p* < 0.001).

To evaluate the prognostic value and determine the best cutoff for the CRP/Alb ratio for predicting disease severity progression among patients with severe COVID-19, receiver operating characteristic curves were generated. The areas under the receiver operating characteristic curves (AUCs) were 0.769 (95% CI: 0.709-0.829, *p* < 0.001) for albumin, 0.838 (95% CI: 0.788-0.888, *p* < 0.001) for CRP, 0.866 (95% CI: 0.822-0.911, *p* < 0.001) for the CRP/Alb ratio, 0.107 (95% CI: 0.060-0.153, *p* < 0.001) for SpO2, and 0.748 (95% CI: 0.671-0.825, *p* < 0.001) for D-dimer levels (Table 5). As shown in Figure 3, the AUC of the CRP/Alb ratio was larger than that of CRP (*Z* statistic: 3.271, *p* = 0.0011) and albumin (*Z* statistic: 3.177, *p* = 0.0015). The best cutoff point for the CRP/Alb ratio was 1.843 for predicting in-hospital progression, with a sensitivity of 91.1% and specificity of 78.0%. Thus, high CRP/Alb ratio was defined as ≥1.843. Furthermore, the Kaplan-Meier curves confirmed that patients with severe COVID-19 who had a high CRP/Alb ratio (≥1.843) had a markedly higher rate of clinical deterioration (Figure 4). The clinical outcomes in the subgroups of patients with severe COVID-19 stratified by the CRP/Alb ratio are listed in Table 6. Patients in the high CRP/Alb ratio group had higher rates of in-hospital death, ICU admission, invasive mechanical ventilation, progression to critical illness, higher disease severity scores, and a longer hospital stay (*p* < 0.001, Table 6).

## 4. Discussion

Severe COVID-19 can easily progress to critical illness or death, and the management of these cases has become a challenge in the face of the COVID-19 outbreak. Clinicians urgently need reliable biomarkers related to disease progression in patients with severe COVID-19 to stratify high-risk patients; these can ensure early individualized treatment and the best allocation of limited medical resources to reduce serious complications and death. In this study, we found a high CRP/Alb ratio in progressive patients and demonstrated that the CRP/Alb ratio on admission had the strongest positive correlation with the SOFA score and length of hospital stay in survivors. Moreover, SpO2, D-dimer levels, and CRP/Alb ratio on admission were independent predictors of progression to critical disease or death in patients with severe COVID-19. A higher CRP/Alb ratio (≥1.843) was closely associated with higher rates of hospital mortality, ICU admission, invasive mechanical ventilation, and a longer hospital stay.

The clinical course, morbidity, and mortality of COVID-19 show significant differences worldwide. In our cohort, the in-hospital mortality rate of patients with COVID-19 was 7.3%. However, previous large-scale multicenter clinical epidemiological studies have shown that in the early stages of the COVID-19 pandemic, the mortality rate of patients with COVID-19 in China was 1.4–3.1% [3, 19], which was lower than that in our cohort. The reason for these differences may be related to the high proportion of patients with severe COVID-19 in our cohort. In addition, in the early stages of the COVID-19 outbreak, the overall case fatality rate (CFR) was 26% in the UK [20], 15.3% in the United States [21], and 29.7% in Italy [22], which were significantly higher than those in China. The reasons for the discrepancies in mortality among patients with COVID-19 worldwide have not been fully clarified. In addition, older age, male sex, and comorbidities are independent mortality predictors in patients with COVID-19 [21, 22]. Studies have revealed that Italian patients are older than Chinese patients, have a higher proportion of male patients, and have more comorbidities, which may be closely related to the high mortality rate of patients with COVID-19 in Italy [23]. Moreover, the causes of differences in mortality around the world may include genetic factors, government epidemic prevention measures, investment and allocation of health resources, and differences in the ability of health systems to respond to outbreaks.

Patients with severe and critical COVID-19 usually show excessive systemic inflammation and poor nutritional status [4, 24]. Recent studies have shown that combined biomarkers, including PNI [24], SII [17], NLR [25, 26], CRP/Alb ratio [13], and PLR [27], are indicators of systemic nutritional status and inflammation, which are closely related to the prognosis and severity of COVID-19. In previous studies, patients with severe COVID-19 and nonsurvivors had significantly higher NLR, PLR, SII, and CRP/Alb ratio values, but lower PNI values [13, 17, 25–28]. Routine blood examination is a simple, inexpensive, and informative method. In a previous study, red blood cell distribution width, NLR, and platelet count can accurately predict the inhospital mortality of patients with COVID-19, which may help clinicians to formulate monitoring and treatment strategies for patients with COVID-19 [26]. However, these studies focused on the differences in these combined biomarkers between survivors and nonsurvivors or between patients with severe and nonsevere COVID-19, which mixed survivors of severe and critical cases together. This may lead to confusion and obscure the differences between patients with different courses and disease progression. However, it is unclear how the values of these biomarkers change when predicting clinical disease progression in patients with severe COVID-19. In our study, we found that the NLR, PLR, SII, and CRP/Alb ratio were higher in patients with progressive disease than in those with stable disease. Moreover, patients with progressive disease had lower PNI values than those with stable disease. Correlation analysis showed that NLR, PLR, SII, and the CRP/Alb ratio were positively correlated with the SOFA score, CURB-65 score, and length of hospital stay. The CRP/Alb ratio had the strongest positive correlation with the SOFA score and length of hospital stay among survivors. However, multivariate logistic regression analysis showed that among these combined biomarkers, only the CRP/Alb ratio was an independent risk factor for disease progression in patients with severe COVID-19. Therefore, the CRP/Alb ratio may be used as a novel and promising predictor for early risk stratification in patients with severe COVID-19.

Several studies have revealed that in many severe and critically ill patients, SARS-CoV-2 can induce a hyperinflammatory response characterized by excessive release of inflammatory cytokines, which is usually defined as a cytokine storm, with increased production of cytokines such as tumor necrosis factor *α* (TNF-*α*), macrophage inflammatory protein 1A, monocyte chemoattractant protein-1, interleukin (IL)-6, IL-7, and inflammatory chemokines [4, 29]. CRP is an innate acute phase protein produced by the liver and has been used as a systematic marker of tissue injury, infection, and inflammation in clinics. In the blood of healthy people, the level of C-reactive protein is usually very low, and its concentration in the blood can increase markedly and rapidly when there is an acute inflammatory reaction induced by trauma and infection [30]. A high CRP level is not only a key biomarker of disease progression and severity in patients with COVID-19 but is also a risk factor for death in patients with severe COVID-19 and is indicative of cytokine storm syndrome in patients with COVID-19 [7]. Similarly, in our study, patients with progressive disease had higher CRP levels than patients with stable disease.

Albumin is a protein with important physiological functions, such as maintaining plasma colloid osmotic pressure, intravascular transport of some substances, inflammatory reactions, thrombosis, and lipid metabolism [31–33]. Albumin levels are closely related to protein intake and absorption, liver function, and inflammation. Therefore, it is classically regarded as a biomarker associated with malnutrition and poor health. Patients with severe and critical COVID-19 often show poor nutritional status and excessive inflammation [4, 24]. Previous studies have shown that patients with COVID-19 have lower albumin levels, and low levels of serum albumin are significantly related to disease severity and adverse outcomes in patients with COVID-19 [34]. In our study, patients with progressive disease had higher CRP and aspartate aminotransferase levels and lower albumin levels than patients with stable disease. Low albumin levels in patients with progressive disease may be associated with impaired liver function and excessive inflammation.

As a new scoring index based on inflammation and nutrition, the CRP/Alb ratio combines CRP and Alb levels. It has been increasingly used in recent years. The CRP/Alb ratio was initially studied as a prognostic indicator of sepsis. The CRP/Alb ratio was found to predict disease severity and 90-day mortality in patients with sepsis [35]. Recently, researchers have used the CRP/Alb ratio to predict the clinical prognosis of patients with cancer. A high CRP/Alb ratio is considered a marker of adverse clinical outcomes in tumors [10, 11]. Likewise, in our study, a high CRP/Alb ratio was closely related to disease severity and progression and was an independent risk factor for disease progression in patients with severe COVID-19. Therefore, the CRP/Alb ratio may represent a balance between inflammation and nutritional status, and an increased CRP/Alb ratio may indicate an increased risk of disease progression in patients with severe COVID-19. In addition, as a biomarker of disease progression in patients with severe COVID-19, the CRP/Alb ratio has the following advantages. First, some inflammatory markers (such as ferritin, TNF-*α*, and IL-6) that were confirmed to be elevated during cytokine storm in patients with severe COVID-19 may not be available in most grassroots hospitals or laboratories in developing countries and are mainly used for research purposes. However, the detection techniques for CRP and Alb are reliable, simple, and cheap; they are usually part of the admission examination in general hospitals, especially in the ICU, and are easy to obtain. Second, previous studies have reported that CRP levels in patients with severe COVID-19 increased significantly in the early stages before lung lesions were found on computed tomography [36]. Third, the AUC of the CRP/Alb ratio was larger than that of albumin or CRP (*p* < 0.01). Therefore, the CRP/Alb ratio is easy to obtain and cheap, changes early with the progression of the disease, and can be used as an effective index for the early detection of disease progression in patients with severe COVID-19.

Our study has a few limitations. First, this was a retrospective study. A large cohort study is needed to further confirm our conclusions. Second, our study did not include mild and moderate cases; therefore, the conclusions of the study may not apply to moderate and mild patients. Third, the study was based on the hospitalization of patients with severe COVID-19 rather than on the onset of symptoms to discharge, which may lack earlier data on CRP and Alb levels.

## 5. Conclusion

In conclusion, our results demonstrated that SpO2, D-dimer levels, and the CRP/Alb ratio on admission were independent risk factors for progression to critical disease or death in patients with severe COVID-19. A high CRP/Alb ratio on admission was associated with disease severity, clinical progression, and poor prognosis of COVID-19. The CRP/Alb ratio may provide clinicians with valuable early prognostic information to facilitate the early risk stratification of disease progression in patients with severe COVID-19 and guide medical staff to monitor and treat patients with a higher risk of disease progression more strictly to improve clinical prognosis.

## Figures and Tables

**Table 1:** Baseline characteristics of patients with stable and progressive COVID-19.

Variables	Total (*n* = 465)	Disease progression		*p* value
		Stable (*n* = 409)	Progressive (*n* = 56)	
Age, median (IQR), years	62.0 (54.0-69.0)	61.0 (53.0-68.0)	67.5 (57.0-73.8)	0.001
Male, *n* [%]	248 (53.3%)	208 (50.9%)	40 (71.4%)	0.004
Comorbidities, *n* [%]				
Hypertension	223 (48.0%)	184 (45.0%)	39 (69.7%)	0.001
Diabetes	102 (21.9%)	89 (21.8%)	13 (23.2%)	0.805
Coronary heart disease	63 (13.5%)	49 (12.0%)	14 (25.0%)	0.008
Chronic obstructive lung disease	37 (8.0%)	27 (6.6%)	10 (17.9%)	0.008
Malignancy	30 (6.5%)	26 (6.4%)	4 (7.1%)	0.822
Vital signs				
Respiration rate, median (IQR),breaths per minute	21.0 (20.0-25.0)	21.0 (20.0-25.0)	23.5 (22.0-29.5)	<0.001
SpO_2_, median (IQR), %	92.0 (90.0-93.0)	92.0 (90.0-93.0)	85.0 (82.3-87.8)	<0.001
Pulse, median (IQR), beats per minute	88.0 (78.5-101.0)	88.0 (78.0-100.5)	92.5 (80.0-104.8)	0.140
MAP, median (IQR), mm Hg	96.7 (90.0-106.2)	96.7 (90.0-106.0)	96.2 (91.0-106.6)	0.916
Temperature, median (IQR), °C	36.8 (36.4-37.3)	36.8 (36.3-37.3)	37.0 (36.5-37.8)	0.010
Disease severity scores, median (IQR)				
SOFA score	2.0 (2.0-3.0)	2.0 (2.0-3.0)	4.0 (3.0-4.0)	<0.001
CURB-65 score	1.0 (0.0-1.0)	1.0 (0.0-1.0)	1.0 (1.0-2.0)	<0.001
Clinical outcomes				
Invasive mechanical ventilation, *n* [%]	43 (9.2%)	0 (0.0%)	43 (76.8%)	<0.001
ICU admission, *n* [%]	40 (8.6%)	0 (0.0%)	40 (71.4%)	<0.001
Length of hospital stay of survivors, median (IQR), days	31.0 (22.0-40.0)	31.0 (22.0-38.0)	51.0 (45.0-55.0)	<0.001
Hospital mortality, *n* [%]	34 (7.3%)	0 (0.0%)	34 (60.7%)	<0.001

Data are presented as medians (IQR) and *n* (%). *p* values were calculated by Student *t*-test, Mann–Whitney *U* test, Chi-square test, or Fisher's exact test, as appropriate. *p* values indicate differences between the stable and progressive patients. Abbreviations: IQR: interquartile range; SpO_2_: percutaneous oxygen saturation; MAP: mean arterial pressure; SOFA score: Sequential Organ Failure Assessment score.

**Table 2:** Laboratory findings in patients with stable and progressive COVID-19 on admission.

Laboratory findings	Normal range	Total (*n* = 465)	Disease progression		*p* value
			Stable (*n* = 409)	Progressive (*n* = 56)	
Blood routine					
White blood cell count, ×10^9^/L	3.5-9.5	6.0 (4.4-7.8)	5.8 (4.4-7.3)	8.8 (4.7-11.9)	<0.001
Platelet count, ×10^9^/L	125-350	223.0 (169.0-293.0)	232.0 (177.0-301.0)	165.0 (110.5-220.3)	<0.001
Neutrophil count, ×10^9^/L	1.8-6.3	4.0 (2.9-6.2)	3.9 (2.8-5.5)	7.5 (3.8-10.3)	<0.001
Lymphocyte count, ×10^9^/L	1.1-3.2	1.0 (0.7-1.4)	1.1 (0.8-1.5)	0.6 (0.4-0.8)	<0.001
Hemoglobin, g/L	115-150	125.0 (114.0-136.0)	125.0 (113.0-136.0)	128.0 (115.0-141.5)	0.239
Blood biochemistry					
Aspartate aminotransferase, U/L	8-40	29.0 (22.0-42.0)	28.0 (21.0-41.0)	39.5 (28.0-63.8)	<0.001
Alanine aminotransferase, U/L	5-35	32.0 (20.5-52.0)	32.0 (20.0-51.0)	39.0 (24.3-63.0)	0.105
Lactate dehydrogenase, U/L	109-245	256.0 (196.0-353.5)	240.0 (191.5-319.5)	399.5 (323.8-619.8)	<0.001
Total bilirubin, *μ*mol/L	3.0-20	10.9 (8.3-15.1)	10.7 (8.0-14.6)	12.4 (9.8-20.2)	0.003
Albumin, g/L	33-55	31.3 (27.5-34.8)	31.7 (28.3-35.7)	26.8 (22.8-30.6)	<0.001
Blood urea nitrogen, mmol/L	2.9-8.2	4.8 (3.7-6.6)	4.7 (3.6-6.0)	6.5 (4.2-9.1)	<0.001
Creatinine, *μ*mol/L	41-81	68.7 (57.4-81.3)	68.0 (56.8-80.3)	74.6 (65.6-87.4)	0.020
Myocardial injury mediators					
Creatine kinase, U/L	24-170	68.0 (43.0-124.5)	66.0 (42.0-117.0)	105.0 (48.5-259.8)	0.001
High-sensitive cardiac troponin I, ng/L	<26.2	4.2 (2.2-11.0)	3.7 (2.0-8.0)	16.5 (7.5-95.3)	<0.001
Inflammatory mediators					
C-reactive protein, mg/L	0-8	30.8 (4.3-66.1)	22.0 (3.9-57.9)	100.2 (51.8-131.0)	<0.001
Blood coagulation					
D-dimer, *μ*g/mL	0-0.5	0.7 (0.3-2.0)	0.6 (0.3-1.6)	5.5 (0.9-8.0)	<0.001
Prothrombin time, s	11.0-16.0	13.1 (12.5-14.1)	13.0 (12.5-13.8)	14.1 (13.0-14.9)	<0.001
International normalized ratio	0.83-1.36	1.0 (1.0-1.1)	1.0 (1.0-1.1)	1.1 (1.0-1.2)	<0.001
Bacterial infection mediators					
Procalcitonin, *μ*g/L	<0.05	0.1 (0.1-0.1)	0.1 (0.1-0.1)	0.2 (0.1-0.4)	<0.001
Lipids					
TC, mmol/L	0-5.2	4.1 (3.5-4.7)	4.1 (3.5-4.7)	3.9 (3.2-4.6)	0.083
TG, mmol/L	0-1.7	1.3 (1.0-1.8)	1.3 (1.0-1.8)	1.2 (1.1-1.7)	0.636
HDL-C, mmol/L	1.1-1.74	0.9 (0.8-1.1)	0.9 (0.8-1.1)	0.8 (0.6-0.9)	<0.001
LDL-C, mmol/L	0-3.12	2.4 (1.9-2.9)	2.4 (1.9-2.9)	2.3 (1.9-2.9)	0.651
apoA-I, g/L	1-1.6	0.8 (0.7-1.0)	0.8 (0.7-1.0)	0.7 (0.6-0.8)	<0.001
apoB, g/L	0.6-1.2	0.9 (0.8-1.1)	0.9 (0.8-1.1)	1.0 (0.8-1.2)	0.204
Lp(a), mg/dL	0-30	14.1 (6.3-25.8)	13.7 (5.8-24.7)	16.3 (8.4-36.3)	0.030

Data are presented as medians (IQR). *p* values were calculated by Student *t*-test and Mann–Whitney *U* test, as appropriate. *p* values indicate differences between the progressive and stable patients. Abbreviations: TC: total cholesterol; TG: triglycerides; HDL-C: high-density lipoprotein cholesterol; LDL-C: low-density lipoprotein cholesterol; apoA-I: apolipoprotein A-I; apo-B: apolipoprotein B; Lp(a): lipoprotein A; CRP: C-reactive protein.

**Table 3:** Correlations among new serological indicators, disease severity scores, and length of hospital stay in survivors.

	Statistics	SOFA score	CURB-65 score	Length of stay of survivors
NLR	*r*_*s*_	0.437	0.434	0.421
	*p*	<0.001	<0.001	<0.001
PLR	*r*_*s*_	0.157	0.185	0.281
	*p*	0.001	<0.001	<0.001
PNI	*r*_*s*_	-0.280	-0.290	-0.421
	*p*	<0.001	<0.001	<0.001
SII	*r*_*s*_	0.232	0.310	0.344
	*p*	<0.001	<0.001	<0.001
CRP/Alb ratio	*r*_*s*_	0.661	0.415	0.552
	*p*	<0.001	<0.001	<0.001

Correlations between variables were analyzed with Spearman's coefficients. Abbreviations: NLR: neutrophil-lymphocyte ratio; PLR: platelet-lymphocyte ratio; PNI: prognostic nutritional index; SII: systemic immune-inflammation index; CRP: C-reactive protein; Alb: albumin; SOFA score: Sequential Organ Failure Assessment score.

**Table 4:** Logistic regression analysis of risk factors for clinical progression in patients with severe COVID-19.

	Univariate analysis		Multivariate analysis	
Variables	OR(95% CI)	*p* value	OR (95% CI)	*p* value
Age	1.040 (1.015-1.066)	0.002		
Male	2.416 (1.311-4.452)	0.005		
Hypertension	2.805 (1.536-5.122)	0.001		
Coronary heart disease	2.449 (1.248-4.807)	0.009		
Chronic obstructive lung disease	3.076 (1.400-6.759)	0.005		
Respiration rate	1.116 (1.054-1.181)	<0.001		
SpO_2_	0.706 (0.649-0.768)	<0.001	0.794 (0.726-0.868)	<0.001
Temperature	1.492 (1.105-2.013)	0.009		
NLR	1.132 (1.089-1.178)	<0.001		
PLR	1.002 (1.000-1.003)	0.045		
PNI	0.979 (0.973-0.986)	<0.001		
SII	1.000 (1.000-1.001)	<0.001		
CRP/Alb ratio	2.176 (1.815-2.610)	<0.001	1.888 (1.516-2.352)	<0.001
Aspartate aminotransferase	1.013 (1.006-1.021)	0.001		
Lactate dehydrogenase	1.007 (1.005-1.009)	<0.001		
Total bilirubin	1.083 (1.042-1.124)	<0.001		
Creatine kinase	1.003 (1.001-1.004)	<0.001		
Blood urea nitrogen	1.055 (1.009-1.104)	0.019		
D-dimer	1.411 (1.286-1.548)	<0.001	1.219 (1.085-1.370)	0.001
Prothrombin time	1.318 (1.126-1.543)	0.001		

Abbreviations: SpO_2_: percutaneous oxygen saturation; NLR: neutrophil-lymphocyte ratio; PLR: platelet-lymphocyte ratio; PNI: prognostic nutritional index; SII: systemic immune-inflammation index; CRP: C-reactive protein; Alb: albumin.

**Table 5:** Receiver operating characteristic curves of Alb, CRP, CRP/Alb ratio, SpO2, and D-dimer levels to predict disease severity progression.

Parameter	AUC (95% CI)	SE	*p* value
Alb	0.769 (0.709-0.829)	0.031	<0.001
CRP	0.838 (0.788-0.888)	0.025	<0.001
CRP/Alb ratio	0.866 (0.822-0.911)	0.023	<0.001
SpO2	0.107 (0.060-0.153)	0.024	<0.001
D-dimer	0.748 (0.671-0.825)	0.039	<0.001

Abbreviations: SpO2: percutaneous oxygen saturation; CRP: C-reactive protein; Alb: albumin; AUC: area under the ROC curve; SE: standard error.

**Table 6:** Patients' clinical outcomes and disease severity scores grouped according to the CRP/Alb ratio.

Variable	^a^High CRP/Alb ratio (*n* = 141)	^b^Low CRP/Alb ratio (*n* = 324)	*p* value
Hospital mortality, *n* [%]	29 (20.6%)	5 (1.5%)	<0.001
ICU admission, *n* [%]	35 (24.8%)	5 (1.5%)	<0.001
Invasive mechanical ventilation, *n* [%]	38 (27.0%)	5 (1.5%)	<0.001
Progress to critical COVID-19, *n* [%]	50 (35.5%)	6 (1.9%)	<0.001
Length of hospital stay of survivors, days	40.0 (34.0-47.8)	29.0 (21.0-35.0)	<0.001
SOFA score	3.0 (3.0-4.0)	2.0 (2.0-3.0)	<0.001
CURB-65 score	1.0 (1.0-2.0)	1.0 (0.0-1.0)	<0.001

Data are presented as medians (IQR) and *n* (%). *p* values were calculated by Mann–Whitney *U* test, Chi-square test, or Fisher's exact test, as appropriate. *p* values indicate differences between the high CRP/Alb ratio group and low CRP/Alb ratio group. Abbreviations: ICU: intensive care unit; CRP: C-reactive protein; Alb: albumin; SOFA score: Sequential Organ Failure Assessment score. ^a^CRP/Alb ratio ≥ 1.843. ^b^CRP/Alb ratio < 1.843.

## Data Availability

The datasets used and/or analyzed during the current study are available from the corresponding author (Pinhua Pan, Phone: +86-0731-89753287, E-mail: pinhuapan668@csu.edu.cn) on reasonable request.
